# *Pseudomonas aeruginosa* orbital cellulitis complicated by ophthalmic artery occlusion in an immunocompetent patient: A case report

**DOI:** 10.1016/j.amsu.2021.102791

**Published:** 2021-09-05

**Authors:** Ben Abdesslem Nadia, Doukh Meriem, Mahjoub Ahmed, Mahjoub Anis, Ghorbel Mohamed, Mahjoub Hechemi, Krifa Fethi, Knani Leila

**Affiliations:** Department of Ophthalmology, Farhat Hached Hospital, Sousse, Tunisia

**Keywords:** Pseudomonas aeruginosa, Orbital cellulitis, Ophthalmic artery occlusion, Immunocompetent adult, Case report

## Abstract

**Introduction:**

We described a case of a pseudomonas aeruginosa subperiosteal abscess in a healthy adult, complicated by ophthalmic artery occlusion.

**Case presentation:**

A 41-year-old woman presented with the chief complaint of a severe painful left eyelid. The visual acuity was limited to light perception. Fundus examiantion showed diffuse retinal edema, papillary swelling and whitened retinal vessels without cherry-red spot. Multimodal imaging confirmed the diagnosis of ophthalmic artery occlusion. Computed tomography study was performed and objectified a pansinusitis complicated by left orbital cellulitis and a 7.4mm × 29.8 mm subperiosteal abscess (SPA). In addition to intravenous antibiotics, surgical drainage of the SPA was performed. The bacterial culture of the abscess has shown growth of *Pseudomonas aeruginosa* and laboratories studies did not find any cause of immunodeficiency. Although medical and surgical treatment, the retinal damage was irreversible with visual acuity limited to light perception.

**Clinical discussion:**

The developing of a subperiosteal abscess (SPA) of the orbit is a serious complication that arises usually from bacterial sinusitis and can lead to sight threatening complications. *Pseudomonas aeruginosa* is not common in healthy adults. An early diagnosis and an adequate treatment are important for the visual prognosis.

**Conclusion:**

Orbital cellulitis should be diagnosed and treated promptly, even in healthy people, to improve visual prognosis.

## Highlights

• The developing of a subperiosteal abscess of the orbit is a serious complication of bacterial sinusitis.
• Orbital cellulitis incriminating Pseudomonas species are very rare.
• Nowadays, the severe cases resulting from orbital cellulitis have dropped significantly due to the general use of antibiotics.
• Orbital cellulitis are sight threatening, even in immunocompetent people.

## 1 Introduction

Orbital cellulitis (OC) is an infection involving ocular tissues posterior to orbital septum [1]. It's an ophthalmic emergency that results most commonly in spreading infection from the paranasal sinuses [2]. The majority of studies have found that *Staphylococcus aureus* and Streptococcus species are the most common pathogens [3]. Although, OC incriminating Pseudomonas species are very rare [1]. It is implicated in 7% of cases [4].

The developing of a subperiosteal abscess (SPA) of the orbit is a serious complication that arises usually from bacterial sinusitis and can lead to sight threatening complications [5]. Therefore, an early diagnosis and an adequate treatment are important for the visual prognosis.

To the best of our knowledge, this is, conceivably, the first case reporting a *Pseudomonas aeruginosa* subperiosteal abscess in a healthy adult, complicated by ophthalmic artery occlusion.

## 2 Case presentation

A 41-year-old Arabic woman presented with the chief complaint of a severe painful left eyelid swelling without any prior systemic or ocular history, apart from an occasional use of nasal corticosteroid spray for allergic rhinitis.

**Fig. 1:**
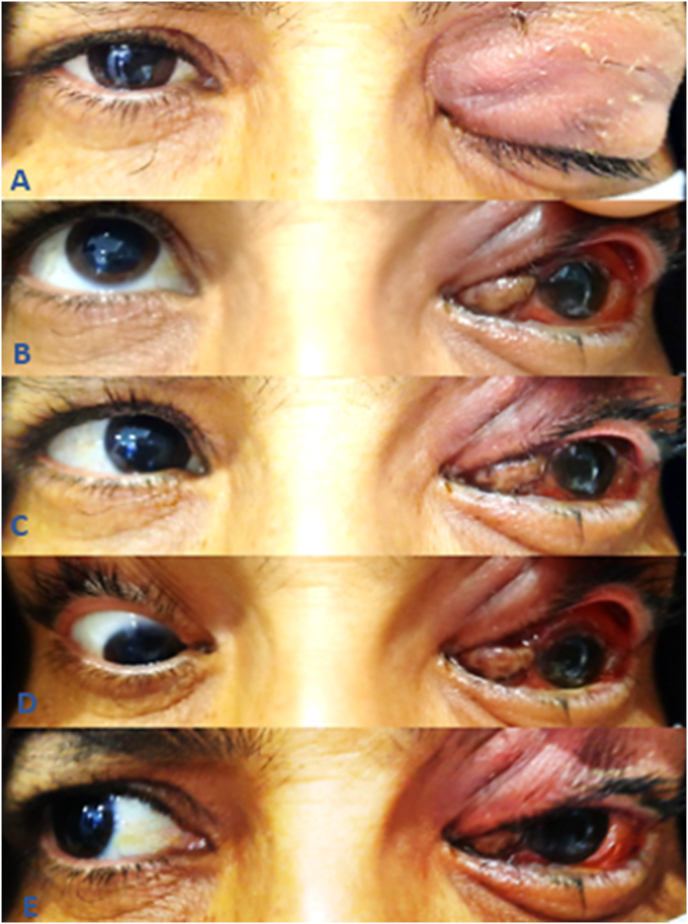
Photography of the patient (A) Primary position, showing eyelid edema with proptosis of the left eye. (B) up gaze, (C) left gaze, (D) down gaze, and (E) right gaze showing ophthalmoplegia with an important chemosis.

**Fig. 2:**
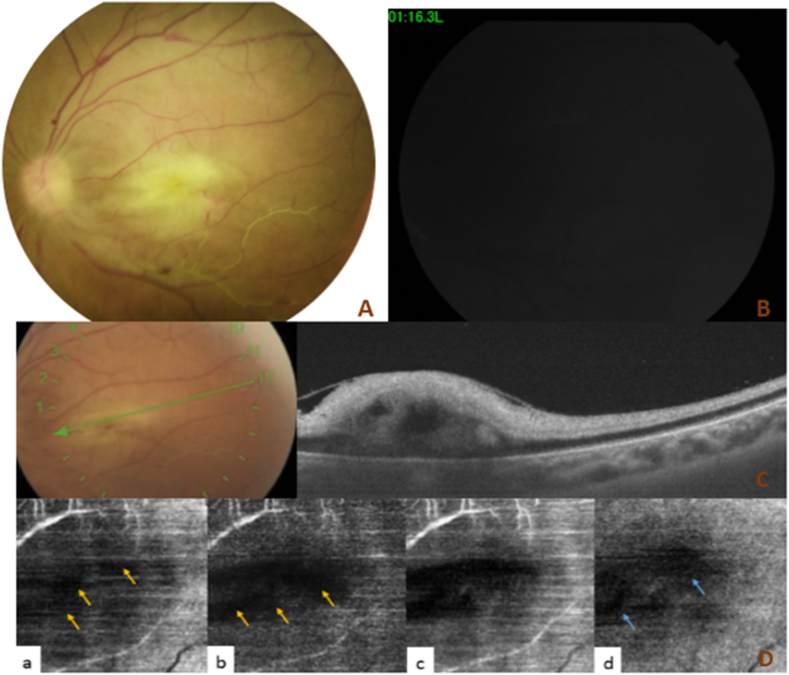
Multimodal imaging of the left eye A: Fundus photography showing a diffuse retinal whitening, papillary edema, whitened retinal vessels without cherry-red spot. B: Fluorescein angiography, showing an almost complete absence of flow in the posterior pole at 1 minute 16 seconds. C: Macular SD-OCT, showing a macular retinal thickening with hyper-reflectivity of the inner retinal layers. D: A 4.5*4.5 optical coherence tomography angiography of the left eye, showing extensive areas of hyposignal (yellow arrows) in superficial capillary plexus (a) and deep capillary plexus (b). The choriocapillaris slab (d), showing large areas of hyposignal (blue arrows). (For interpretation of the references to colour in this figure legend, the reader is referred to the Web version of this article.)

Our ophthalmological examination found in the left eye a visual acuity (VA) limited to light perception, the eyelid was markedly swol1en and erythematous, a severe hemorrhagic chemosis, a proptosis with an ophtalmoplegia (Fig. 1) and a non-reactive semi-mydriasis. The fundus examination was difficult and it revealed a diffuse retinal whitening, papillary edema, tortuosity of the retinal veins, whitened retinal vessels without cherry-red spot (Fig. 2A). The examination of the right eye was normal.

The intraocular pressure IOP was 12 mmHg in right eye and couldn't be evaluated in the left eye.

Fluorescein angiography of the left eye showed an almost complete absence of retinal and choroidal flow and thus, confirmed the diagnosis of ophthalmic artery occlusion (Fig. 2B). Macular Spectral domain optical coherence tomography (SD-OCT) revealed macular retinal thickening with hyper-reflectivity of the inner retinal layers (Fig. 2C). The optical coherence tomography angiography (OCT-A) of the left eye demonstrated extensive areas of capillary non-perfusion in both superficial capillary plexus and deep capillary plexus with large areas of intense hyposignal in the choroid layer (Fig. 2D).

**Fig. 3:**
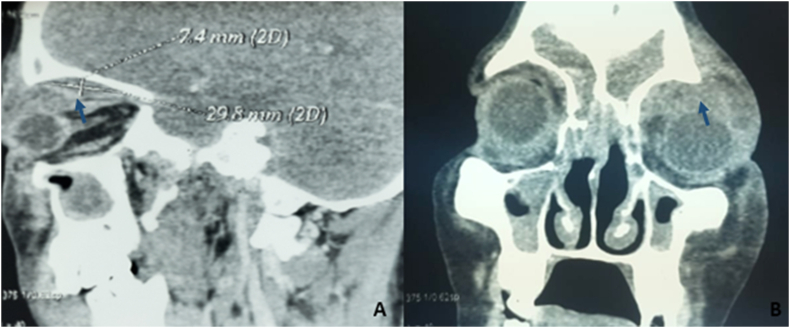
A CT-scan, showing a left orbital cellulitis and a 7.4mm × 29.8mm subperiosteal abscess complicating pansinusitis (blue arrow). A: Sagittal view, B: Frontal view. (For interpretation of the references to colour in this figure legend, the reader is referred to the Web version of this article.)

Then, a computed tomography study was performed and objectified a pansinusitis complicated by left orbital cellulitis and a 7.4mm × 29.8 mm subperiosteal abscess (SPA) (Fig. 3).

Intravenous antibiotics were promptly delivered combining cefotaxime 1.5 g every 6 hours, vancomycin 1 g every 12 hours and metronidazole 500 mg every 8 hours and a surgical drainage of the SPA was planned in 6 h under general anesthesia because of the visual impairment.

A sub-brown incision was made in the skin. The periosteum was identified at the orbital rim and incised with a 15 steel surgical blade. The subperiostal space was entered and a dissection was carried down posteriorly until the abscess cavity was objectified. The pus was aspired and sent for microbiological exam and culture. A drain was placed for 2 days and the wound was closed in layers.

Oral and topical anti-glaucoma drugs including Timolol 1 drop every 12 hours and Acetazolamide 250mg 4 times per day, were also prescribed.

The bacterial culture of the abscess has shown growth of *Pseudomonas aeruginosa* (Table 1) and antibiotic therapy was switched: Ciprofloxacin 200 mg every 12 hours and ceftazidime 2000 mg once a day. A brain magnetic resonance imaging was performed to rule out cavernous sinus thrombosis.

**Table 1:** Antibiotic sensitivity profile of bacterial pathogen: *Pseudomonas aeruginosa*

Antibiotics	Result
Amikacin	sensitive
Ceftazidime	intermediate
Ciprofloxacin	sensitive
Colistine	sensitive
Gentamicin	sensitive
Imipenem	sensitive
Piperacillin	sensitve
Piperacillin-tazobactam	resistant
Ticarcillin	sensitive
Ticarcillin-clavulanic acid	resistant
Tobramycin	sensitive

Laboratory studies, including complete blood count and Human Immunodeficiency Virus HIV serology, revealed no cause of immunodeficiency.

At fifteen days follow up, we noted resolution of chemosis and eyelid edema but the visual loss remained. The fundus examination of the left eye revealed reperfusion of the superior retina vessels, presence of sheathed vessels in the inferior retina and diffuse disc pallor. The macular SD-OCT demostrated diffuse retinal thinning with hyper-reflectivity of the inner retinal layers and the OCT-A showed extensive areas of capillary non-perfusion in both superficial capillary plexus and deep capillary plexus.

The patient was recently seen at 3 months of follow up. The visual acuity was always the same. On fundus examination, we noted significant retinal ischemia with fibrosis and optical disc pallor. The SD-OCT peripapillary retinal nerve fiber layer RNFL measures confirmed the optic atrophy. The macular OCT-SD demonstrated retinal thinning with posterior vitreous detachment.

Ophthalmological and otorhinolaryngological exams were planned every 3 months.

This case report has been reported in line with the SCARE Criteria [6].

## 3 Discussion

Sinusitis are common in daily practice and frequently treated without complications. In some cases, it can cause serious orbital complications leading to blindness and even death [5]. The subperiosteal abscess may occur in 42% of cases as complication of sinus disease [1]. In many studies, the ethmoid sinus and also the maxillary sinus are the most common source of infection [[5], [7]]. In our case, the patient has a pansinusitis which is more common in the *Pseudomonas aeruginosa* (PA) sinusitis [8].

In a recent study published in 2017 in Taiwan, the most implicated pathogens was coagulase-negative Staphylococcus spp. (25.3%) and *Staphylococcus aureus* (20.5%) [4]. *PA* is implicated in only 7.2% of cases [4]. In japan, Suzuki reported that *PA* sinusitis rates are increasing [9]. Moreover, only few articles have reported PA causing severe orbital complications and it was about cases of orbital apex syndrome [[10], [11], [12], [13]]. It's noteworthy that most cases have mixed microbial forms and it infects more commonly immunocompromised persons. However, our present case is an immunocompetent woman. The local corticosteroid therapy may reduce the activity of the immune system.

The diagnosis of subperiostal abscess is clinically evident by the visual impairment, directional proptosis, eye pain and chemosis.

CT scan with contrast infusion, including axial and coronal views, is essential to determine the extend of the abscess and to rule out peridural and parenchymal brain abscess.

Complete Blood count show elevated white blood cells WBC. In cases of Pseudomonas cellulitis with eyelid necrosis, it is crucial to research neutropenia. Early recognition with reversal of neutropenia is crucial in the management of this infection.

The possible pathophysiology mechanism of ophthalmic artery occlusion (OAO) noted in our case is the acute elevation of the intraorbital pressure (IOP) due to the rapid progression of SPA in the orbit which is a limited space and so it can hamper the ophthalmic artery circulation. Also, the cherry-red spot is absent because of both retinal and choroidal insufficiency.

Proctor [14] described a case of OC complicated by central retinal artery (CRA) occlusion and explained this by an intraconal mechanical compression of the CRA.

Okamoto [15] reported a case of subperiosteal abscess of the orbit with central retinal artery occlusion with negative microbial cultures and highlighted the poor prognosis associated with SPA which can disturb ophthalmic artery circulation by the elevated intraorbital pressure, causing a direct compressive optic neuropathy or ischemic neuropathy. Keorochana [3] presented a rare case of combined central retinal vein, central retinal artery and cilioretinal artery occlusion with ischemic macular hole secondary to severe *Staphylococcus aureus* OC after black fly bite leading to permanent visual loss.

The OAO complicating OC is a rare condition and has a worse prognosis because of retinal and choroidal involvement. In 1998, a case of ophthalmic artery occlusion following orbital inflammation was reported [16]. Hatipoglu [17] described a more complicated case including ophthalmic artery, cavernous sinus, and superior ophthalmic vein occlusion in a rhino-orbital mucormycosis demonstrated by magnetic resonance imaging MRI.

For the ophtalmoplegia, in some studies [18] it was explained by cavernous sinus thrombosis which is absent in our case. Thus, extraocular muscles paralysis may be due to increased IOP or severe muscles's inflammation [3].

The treatment of orbital cellulitis include broad-spectrum antibiotic therapy for 14 days. In case of visual impairment, the surgical management of SPA is required because medical treatment alone is often ineffective [19]. The aim is to decrease IOP and so relieve the ophthalmic artery circulation. In fact, OAO is a true therapeutic emergency because every minute of delay can be potentially devastating and lead to permanent visual loss [20].

A follow-up of the patient is crucial to evaluate response to medical and surgical treatment and to research any ophthalmological or cerebral complication. The visual and vital prognosis depends on the moment and on the quality of treatment.

In our case, we ignore the exact onset time of SPA and OAO so despite the early surgical intervention, the retinal damage was irreversible.

## 4 Conclusion

Nowadays, the severe cases resulting from OC have dropped significantly due to the general use of antibiotics. However, an early diagnosis of orbital complications and a prompt treatment can improve visual prognosis. Our case demonstrated the retinal damage caused by the PA orbital cellulitis in a healthy adult, supporting by multimodal imaging.

## Sources of funding

None.

## Ethical approval

Not applicable.

## Consent

Written informed consent was obtained from the patient for publication of this case report and accompanying images. A copy of the written consent is available for review by the Editor-in-Chief of this journal on request.

## Author's contribution

Mahjoub Ahmed and Mahjoub Anis collected data, Ben Abdesslem Nadia and Doukh Meriem analysed data and drafted the manuscript. Mahjoub Hechemi, Leila Knani, Ghorbel Mohamed and Krifa Fethi provided critical manuscript revisions.

All authors read and approved the final manuscript.

## Trial registry number

Not applicable.

## Guarantor

Dr Ben Abdesslem Nadia.

## Provenance and peer review

Not commissioned, externally peer reviewed.

## Declaration of competing interest

None.
